# Serological exposure of spotted fever group *Rickettsia* in capybaras (*Hydrochoerus hydrochaeris*) from urban parks in Campo Grande, Brazilian Midwest

**DOI:** 10.1590/0037-8682-0192-2022

**Published:** 2022-09-19

**Authors:** João Bosco Vilela Campos, Filipe Santos Martins, Gabriel Carvalho de Macedo, Wanessa Teixeira Gomes Barreto, Carina Elisei de Oliveira, Amália Regina Mar Barbieri, Marcelo Bahia Labruna, Luiz Gustavo Rodrigues Oliveira-Santos, Heitor Miraglia Herrera

**Affiliations:** 1 Universidade Católica Dom Bosco, Pós-Graduação em Ciências Ambientais e Sustentabilidade Agropecuária, Campo Grande, MS, Brasil.; 2 Universidade Federal de Mato Grosso do Sul, Pós-Graduação em Ecologia e Conservação, Campo Grande, MS, Brasil.; 3 Universidade de São Paulo, Departamento de Medicina Veterinária Preventiva e Saúde Animal, São Paulo, SP, Brasil.

**Keywords:** Capybaras, *Rickettsia* spp., Urban parks, One health, Brazilian mildest

## Abstract

**Background:**

*Rickettsia* of the spotted fever group (SFG) has been reported in ticks and domestic animals in Campo Grande (CG), Midwest Brazil.

**Methods:**

We searched *for Rickettsia* in the SFG in capybaras and their ticks in an urban park in the CG.

**Results:**

The seropositivity rate was 88.2% (15/17). Although 87.7% of the capybaras sampled showed infestations with *Amblyomma sculptum*, *A*. *dubitatum,* and *Amblyomma* spp., no molecular results were detected in ticks.

**Conclusions:**

Since *Rickettsia* from the SFG circulates among capybaras in the urban parks of Campo Grande, this large rodent species should be monitored within the One Health Agenda.

*Rickettsia rickettsii* is the etiological agent of Brazilian spotted fever, an emerging zoonosis of great public health importance^1^. Campo Grande (CG), the capital of Mato Grosso do Sul (MS) state, Brazil, has approximately one million inhabitants. This city has several urban parks, green areas, and conservation units formed by cerrado *sensu stricto* (savanna), cerradão (woodland savanna), and riparian forest^2^. *Rickettsia*, belonging to the spotted fever group (SFG), has been found in CG and its surroundings, showing molecular evidence for *R. parkeri* in *A. sculptum* ^3^, *R. parkeri* strain Atlantic rainforest in *A. ovale* ^4^, *R. parkeri*, *R. africae*, and *R. sibirica* in *A. dubitatum* ^5^. Furthermore, Campos et al.^6^ reported a general seroprevalence of *Rickettsia* spp. in 25.6% of horses sampled in the CG (n=262); 19.8% were exposed to *R. rickettsii*, 16.7% to *R. parkeri*, and 17.5% to *R. amblyommatis*. 

Caviomorph rodent capybaras (*Hydrochoerus hydrochaeris*) play a central role in the epidemiology of *Rickettsia* in urban areas, as they have high reproduction rates and continually maintain active infections in vector ticks^7^ ^,^ ^8^ ^,^ ^9^. Indeed, after primary infection, young animals have high rates of bacteremia^8^. In addition, their extraordinary adaptation to urban areas results in high population densities^2^ ^,^ ^7^. In CG, capybaras typically rest inside forest patches during the day, moving to open grasslands to graze in the twilight, and spending the night in these open areas^2^, thus, playing an important role in dispersing ticks between forest and grassland areas. In urban parks in CG, humans are acclimatized to capybaras, approaching them, walking near them, and spending their daytime recreational time in the same pastures grazed by capybaras at night^2^. 

The scenario found in urban fragments areas in CG was as follows: (a) circulation of *Rickettsia* from the SFG group, (b) high density of capybaras, and (c) spatial sharing between humans and capybaras. This scenario raises a red flag concerning the possibility of rickettsia transmission from the SFG group of capybaras to humans. Therefore, it is necessary to investigate the presence of *Rickettsia* circulating in urban capybaras, mainly those living within urban parks, for the adequate surveillance and epidemiological control of spotted fever in large cities in Brazil. Therefore, this study aimed to investigate the serological occurrence of *Rickettsia* spp. belonging to SFG in capybaras from urban parks in the CG.

This study was performed in two urban areas of CG: (i) the Indigenous Nations Park (PNI) and (ii) the Private Reserve of the Federal University of Mato Grosso do Sul. Between May 2017 and August 2018, 17 capybaras were treated with tiletamine and zolazepam (Zoletil ®Vibrac) using a rifle ( J.M.DB13 ®Daninject). Ticks parasitizing capybaras were collected after visual inspection for 60 s and identified using previously published dichotomous keys^10^. We used an indirect immunofluorescence antibody test (IFAT) to detect IgG antibodies against *Rickettsia* spp. in the SFG according to Campos et al^6^. We used slides containing crude antigens derived from *Rickettsia* isolates from *R. rickettsii* strain Taiacu, *R. parkeri* strain At24, and *R. amblyommatis* strain Ac37, which are available at the Laboratory of Parasitic Diseases (University of São Paulo, Department of Preventive Veterinary Medicine and Animal Health). All field procedures and laboratory studies were conducted under a license granted by the Instituto Chico Mendes de Conservação da Biodiversidade (license number 70946-3). This study was approved by the Ethics Committee for Animal Use at the Universidade Católica Dom Bosco (license number 013/2020).

Our results showed that 88.2% (15/17) of sampled capybaras were seropositive for *Rickettsia* spp. Among these, 64.7% (11/17) were *R. rickettsii*, 88.2% (15/17) were *R. parkeri,* and 41.1% (7/17) were *R. amblyommatis*. We observed that six animals displayed seropositivity for *R. rickettsii* and *R. parkeri*, two for *R. parkeri* and *R*. *amblyommatis*, and five all three species (*R. rickettsia*, *R. parkeri*, and *R*. *amblyommatis*). Only two animals had a single exposure to *R. parkeri*, and we observed four animals with high IgG antibody titers ranging from 1:512 to 1:2048 (Table 1). Moreover, 88.2% (15/17) of the sampled capybaras were infested with ticks (n=80), including 25 specimens of *A. dubitatum* (19 males and 6 females), 29 specimens of *A. sculptum* (16 males and 13 females), and 26 immature forms of *Amblyomma* spp. (24 nymphs and 2 larvae).

**TABLE 1:** End point titers of indirect immunofluorescence assay for three rickettsia species of capybaras (*Hydrochoerus hydrochaeris*) (n=17) sampled in Campo Grande, midwestern Brazil.

Capybara sera	**IFAT titers for the following *Rickettsia* antigens**			PAIHR
	*Rickettsia rickettsii*	*Rickettsia parkeri*	*Rickettsia amblyommatis*	
1	1/128	1/128	NR	
2	NR	1/64	NR	*R. parkeri*
3	NR	NR	NR	
4	NR	1/256	1/128	
5	1/128	1/128	NR	
6	1/128	1/128	NR	
7	1/128	1/128	1/128	
8	NR	1/128	1/256	
9	1/2048	1/2048	1/512	
10	1/256	1/1024	1/256	*R. parkeri*
11	1/256	1/512	NR	
12	1/256	1/256	NR	
13	NR	NR	NR	
14	1/256	1/512	NR	
15	1/128	1/2048	1/1024	
16	1/64	1/256	1/64	*R. parkeri*
17	NR	1/128	NR	*R. parkeri*

**PAIHR:** A possible antigen involved in a homologous reaction (serum showing a *Rickettsia* species titer at least fourfold higher than that observed for any other *Rickettsia* species was considered homologous to the first *Rickettsia* species). **NR:** nonreactive at titer 64 or higher; **IFAT:** indirect immunofluorescence antibody test.

Our results showed that capybaras exposed to *Rickettsia* spp. belonging to the SFG were more widely distributed in the Brazilian Midwest than previously reported^11^. Although we investigated a low number of capybaras, the high seropositivity rates of 88.2% (15/17), with high titers ranging from 1:512 to 1:2048, indicate that capybaras may play an important role in the epidemiology of *Rickettsia* spp*.* in the studied area. Serological confirmation of *Rickettsia* species that may infect capybaras should be observed with caution due to cross-reactions between different rickettsia species belonging to SFG^7^. However, our results showed that the four capybaras sampled were parasitized by *R. parkeri* (Table 1), a species already recorded parasitizing *A*. *dubitatum* in the studied area^5^.

Capybaras are the central host species for Brazilian spotted fever because (i) they develop high rickettsemias (amplifier hosts), ensuring a constant infection of tick vectors, (ii) they have a high reproduction rate, and (iii) they are parasitized by different species of *Amblyomma* ^8^ ^,^ ^12^. Indeed, the high rate of infestation by *A. sculptum*, the main tick vector species for *Rickettsia* spp.^13^, observed in the sampled capybaras suggests a potential risk for transmission of *Rickettsia* spp. Furthermore, since *A. sculptum* has already been reported to parasitize humans^1^ and PNI urban parks are visited daily by hundreds of people^2^, there is a possibility of spillover of *Rickettsia* from the SFG to humans. 

Although *A. dubitatum* has been reported to parasitize *Rickettsia* spp. belonging to the SFG in urban parks in CG^5^, this is the first time this tick species has been found to parasitize capybaras in urban parks in CG, suggesting that *A. dubitatum* may play an important role in the transmission cycles of these rickettsial agents in the study area. Additionally, despite *A. dubitatum* not being a tick species associated with humans^14^, it has been reported that opportunities for pathogen transmission via larvae and nymphs of Amblyomma species are higher in degraded habitats^15^ such as urban parks.

Additionally, capybaras that inhabited urban green areas in the CG presented large home ranges, bimodal daily activity patterns, and remarkable changes in habitat selection throughout the day^2^. Indeed, the wide home ranges, larger than those estimated in natural environments, together with the increase in selectivity patterns for forest areas on days of high human presence reported by Medeiros et al.^2^, strongly favor the spread of ticks infected with *Rickettsia* spp. through urban green areas by capybaras.

We highlight that the capybaras that inhabit the urban parks of CG are the target of constant discussions about translocation to native areas of the Cerrado and Pantanal biomes because of the risk of spillover of zoonotic agents. This topic should be discussed carefully because it has been demonstrated that the introduction of a single infected capybara with at least one infected attached tick is sufficient for the spillover of Brazilian spotted fever in a non-endemic area^12^. 

Campo Grande, in the Midwest region of Brazil, should be monitored since because (i) capybaras that live in urban green areas are highly exposed to *Rickettsia* of the SFG; (ii) these capybaras presented high tick infestations; (iii) the tick species found parasitizing capybaras have anthropophilic habits; (iv) urban green fragments areas of CG have an intense people flow^2^; (v) *A. dubitatum* parasitizing capybaras were found to be infected by *Rickettsia* of the SFG in the studied areas^5^; and (vi) Campos et al.^6^ noticed that 25.6% of 262 sampled horses in the CG were exposed to rickettsia agents of the SFG. Owing to the latent risk of transmission, a surveillance and contingency plan for rickettsioses should be considered for the study area.
